# Identification of a Novel, Oncogenic and Targetable *TPR::ABL2* Fusion Gene in T‐Cell Acute Lymphoblastic Leukaemia

**DOI:** 10.1002/jha2.70255

**Published:** 2026-03-02

**Authors:** Elias Lagonik, Elyse. C. Page, Laura N. Eadie, Caitlin E. Schutz, Jacqueline A. Rehn, Susan L. Heatley, Andrew S. Moore, Muirin Healy, Morag Whyte, Timothy P. Hughes, David T. Yeung, Deborah L. White

**Affiliations:** ^1^ Precision Cancer Medicine Theme South Australian Health and Medical Research Institute Adelaide South Australia Australia; ^2^ Faculty of Sciences, Engineering and Technology The University of Adelaide Adelaide South Australia Australia; ^3^ Faculty of Health and Medical Sciences The University of Adelaide Adelaide South Australia Australia; ^4^ Australian and New Zealand Children's Haematology‐Oncology Group Clayton Victoria Australia; ^5^ Oncology Service Children's Health Queensland Hospital Brisbane Queensland Australia; ^6^ Paediatrics Department Mackay Base Hospital Mackay Queensland Australia; ^7^ Haematology Department Queensland Children's Hospital Brisbane Queensland Australia; ^8^ Haematology Department Royal Adelaide Hospital Adelaide South Australia Australia

**Keywords:** *ABL2*, asciminib, T‐ALL, TKI, TPR

## Abstract

*ABL2* rearrangements represent a subtype of acute lymphoblastic leukaemia (ALL) associated with poor prognosis and survival. This study reports a high‐risk T‐cell ALL (T‐ALL) case with a novel TPR::ABL2 gene fusion resulting from a chromosomal deletion. Overexpression of *TPR::ABL2* in Ba/F3 cells promoted cytokine‐independent growth, demonstrating its oncogenic nature. Both primary patient and Ba/F3 cells carrying *TPR::ABL2* exhibited kinase activation and sensitivity to tyrosine kinase inhibitors (TKIs). This study expands the repertoire of *ABL2* fusions identified in ALL and supports the incorporation of TKIs into T‐ALL treatment regimens to improve outcomes for this subtype.

## 1 Introduction

T‐cell acute lymphoblastic leukaemia (T‐ALL) is an aggressive haematological cancer arising from differentiation‐arrested T‐cell precursors. Next‐generation sequencing has revealed the diverse genomic basis of T‐ALL, which comprises multiple subtypes accounting for 15%–20% of ALL cases [1]. T‐ALL is characterised by high relapse rates, poor outcomes and limited clinically approved targeted treatment options [2, 3]. Current treatment regimens consist of intensive multi‐agent chemotherapy followed by haematopoietic stem cell transplantation (HSCT) [3]. Therefore, there is an unmet need to improve therapeutic approaches. In T‐ALL, recurrent genomic alterations often involve *ABL1* rearrangements, with the *NUP214::ABL1* fusion gene identified in 5%–10% of patients with T‐ALL [3]. The *ABL2* gene, a paralogue of *ABL1*, has also been implicated in T‐ALL, although *ABL2* fusion genes are reported less frequently [1, 3, 4]. Similar to *ABL1*, *ABL2* fusion genes result in the loss of the N‐terminal inhibitory elements, leading to the constitutive activation of the ABL kinase and downstream signalling pathways [5, 6]. *ABL1* and *ABL2* rearrangements (*ABL‐r*) are particularly interesting due to their susceptibility to inhibition by tyrosine kinase inhibitors (TKIs) [7]. In T‐ALL, *ABL‐r* often co‐occur with *TLX3* enhancer hijacking events, resulting in increased expression of the *TLX3* gene [1]. Additional genomic alterations include the activation of signalling pathways (e.g., *NOTCH1*) and the loss of tumour suppressor pathways (e.g., inactivating mutations in *FBXW7* and *CDKN2A/B*) [1, 8]. In this study, we identify the Translocated Promoter Region (*TPR*) gene as a fusion partner of *ABL2* in a paediatric case of T‐ALL. The leukaemogenic potential of the novel *TPR::ABL2* gene and sensitivity to small inhibitors were characterised in vitro and ex vivo.

## 2 Methods and Results

A retrospective genomic analysis was conducted on a cryopreserved bone marrow (BM) sample from a patient at the Queensland Children's Hospital (Brisbane, QLD). Experiments were performed in accordance with the Women's and Children's Health Network Human Research Ethics Committee. See Supporting Information for detailed methodology protocols.

A 6‐year‐old boy presented with a short history of gastrointestinal symptoms, fever and lethargy. Examination revealed cervical lymphadenopathy, hepatosplenomegaly and ascites. The testicular exam was unremarkable. There was mild mediastinal widening on the chest x‐ray, and peripheral blood leukocytosis (white blood cell count of 467.5 × 10^9^/L) with blasts. BM and peripheral blood investigations confirmed a diagnosis of T‐ALL, subsequently shown by genomic analyses to be driven by the novel *TPR::ABL2* fusion gene. There was no CNS involvement. Following induction chemotherapy, residual disease of 8% was demonstrated by flow cytometry. Consolidation was completed as per AALL0434 arm B with nelarabine, and he commenced on dasatinib during this time. End of consolidation testing showed persistent residual disease of 0.08%, so he then received fludarabine, cytarabine, idarubicin (FLA‐IDA) to attain minimal residual disease < 0.01% prior to planned HSCT. He underwent a total body irradiation, cyclophosphamide, thiotepa conditioning and a fully matched unrelated donor peripheral blood HSCT 6 months after initial diagnosis. The procedure was well tolerated with no significant complications, including graft‐versus‐host disease. The patient remains well and in remission, at the time of this report, 2.5 years following the allograft.

**FIGURE 1:**
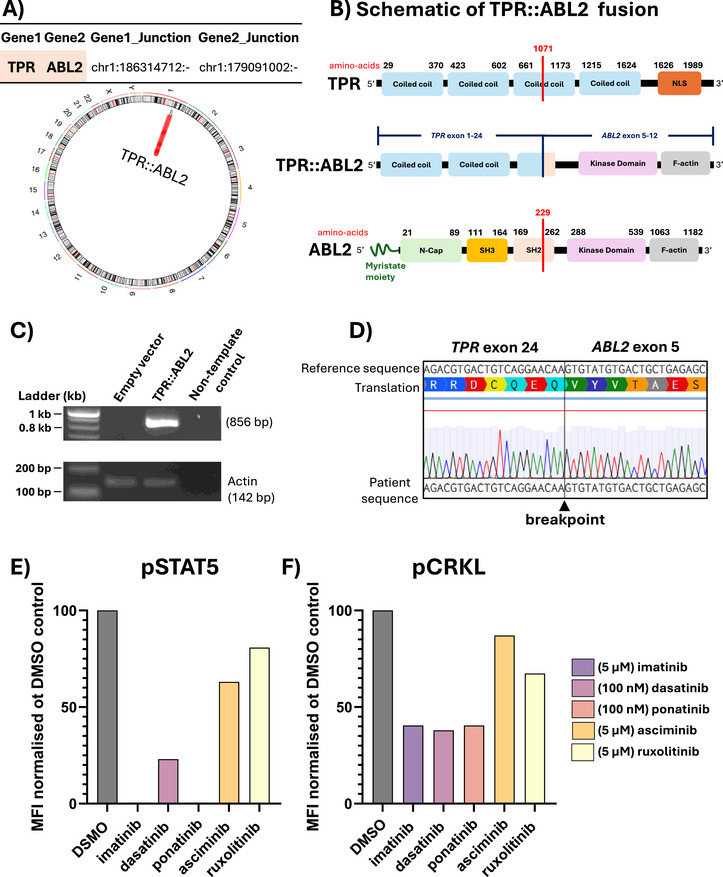
Identification of a novel *TPR::ABL2* fusion gene in T‐cell acute lymphoblastic leukaemia. (A) Circos plot representation of the *TPR::ABL2* fusion gene identified by mRNA‐sequencing of BMMNCs at diagnosis. Isoforms *TPR* (NM_003292) and *ABL2* (NM_001168237). (B) Schematic representation of full‐length *TPR*, full‐length *ABL2*, and *TPR::ABL2* fusion gene. Breakpoints are denoted with a red line. (C) PCR amplification of the *TPR::ABL2* breakpoint region. (D) Sanger sequencing confirmation of the *TPR::ABL2* breakpoint sequence. (E, F) BMMNCs were incubated for 2 h in either vehicle control (DMSO), TKIs, or ruxolitinib (negative control). Phosphorylation of STAT5 and CRKL was assessed by intracellular flow cytometry, compared to isotype controls IgG1‐PE (pSTAT5) or IgG2a‐PE (pCRKL), and normalised to DMSO vehicle control. MFI, mean fluorescence intensity.

Bone marrow mononuclear cells (BMMNCs) were isolated using Lymphoprep density gradient centrifugation. Immunophenotyping of BMMNCs indicated approximately ∼73% lymphoid origin cells. Cells within the lymphoid/blast gate expressed CD3 and CD7 (63%) with negligible expression of CD13, CD33, CD117 (c‐kit) and CD34 (all < 3%) (Figure S1, Table S1). BMMNCs mRNA sequencing revealed a novel cytogenetically cryptic, in‐frame fusion between the 5' end of *TPR* (Exon 1–24, NM_003292) and the 3' end of *ABL2* (Exon 5–13, NM_001168237), resulting from an interstitial deletion on Chr.1q. Breakpoint PCR and Sanger sequencing confirmed the fusion gene, and the complete and partial loss respectively of the ABL2 SH3 and SH2 domain (Figure 1A–D). The novel *TPR::ABL2* fusion gene sub‐classifies this case as *ABL‐*rearranged ALL. While this fusion is novel, other *TPR* fusions have been previously reported in patients with B‐cell and T‐cell ALL [1, 7, 9]. Additional genomic alterations identified in the current patient included the deleterious FBXW7 p.R465C mutation with a variant allele frequency (VAF = 0.29). The FBXW7 protein is a tumour suppressor that regulates NOTCH1 protein activity through direct binding and degradation. The FBXW7 p.R465C mutation results in a loss of function and inability to target NOTCH1 and c‐MYC, a consequence linked to gamma secretase inhibitor resistance [8]. The SALSA Multiplex Ligation‐dependent Probe Amplification (MLPA) assay (#P383, #P202, #P335; MRC‐Holland, Amsterdam, NL) revealed additional high‐risk genomic lesions, including heterozygous deletion of *LEF1* (Exon 1–4; Chr4q25) (Figure S2). Deletions of *LEF1* first exons are frequent in paediatric T‐ALL, although rare in patients with *ABL*rearrangements [1]. MLPA also revealed a focal biallelic deletion of *CDKN2A* (Exon 4; Chr9p21.3) (Figure S2). Deletion of *CDKN2A/B* is observed in the majority of T‐ALL cases [1]. Interestingly, and in contrast to other *ABL*‐rearranged T‐ALL cases, increased expression of *TLX3* was not noted (data not shown) [1]. BMMNCs expressing *TPR::ABL2* demonstrated intrinsically activated phosphorylation of downstream effector proteins, CRKL (pCRKL) and STAT5 (pSTAT5), as measured by intracellular phosphoflow cytometry. TKIs have been used successfully to target *ABL2‐r* in the clinic [7]. ATP‐competitive TKIs, including imatinib, dasatinib and ponatinib, abrogated pCRKL and pSTAT5 signalling, while asciminib and the JAK inhibitor ruxolitinib had minimal effect (Figure 1E,F) (Table S1).

**FIGURE 2:**
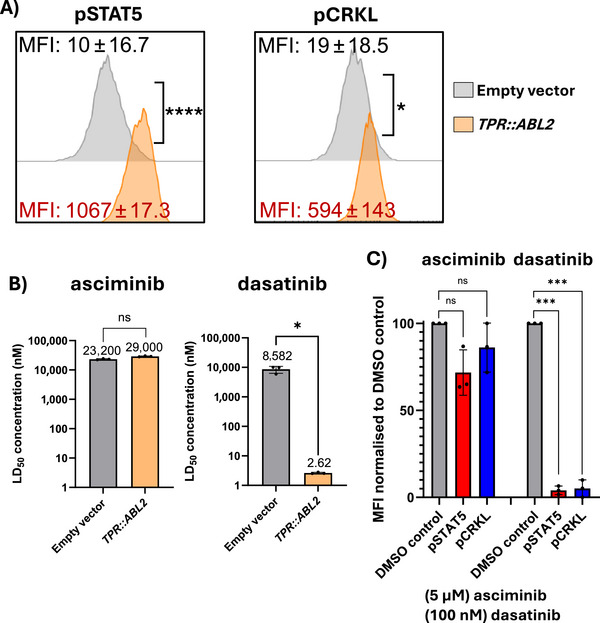
Ba/F3 cells expressing the *TPR::ABL2* fusion gene exhibit constitutive kinase activation and are sensitive to ATP‐competitive TKIs. (A) Cells were assessed for STAT5 and CRKL phosphorylation. Empty vector Ba/F3 cells were cultured in the absence of IL‐3 for 5 h. MFI values of three independent experiments are highlighted. Histograms are representative of *n* = 3 biological replicates. (B) Ba/F3 cells expressing *TPR::ABL2* were incubated for 72 h with vehicle control (DMSO) or increasing concentrations of dasatinib or asciminib. The mean LD_50_ nanomolar concentration for each cell line is denoted above the respective columns. Columns represent mean ± SD. (C) Ba/F3 cells expressing the *TPR::ABL2* were incubated for 2 h in DMSO or 100 nM dasatinib, or 5 µM asciminib. STAT5 and CRKL phosphorylation were assessed by intracellular flow cytometry. Statistical significance was determined by unpaired Student's *t*‐test with Welch's correction (ns =not significant, *p < 0.05, **p < 0.01, ***p < 0.001, ****p < 0.0001). MFI, mean fluorescence intensity.

The full‐length *TPR::ABL2* fusion gene was isolated from patient material (Table S2) and retrovirally transduced into the Ba/F3 cell line. Expression of *TPR::ABL2* resulted in IL‐3‐independent growth, demonstrating constitutive kinase activation and associated phosphorylation of CRKL and STAT5 (Figure 2A). To evaluate the sensitivity of *TPR::ABL2* expressing cells to ATP‐competitive TKIs and the allosteric inhibitor asciminib, cell death assays were conducted (Table S1) and demonstrated the efficacy of dasatinib against the oncoprotein in the nanomolar range (Figure 2B). Asciminib failed to induce cell death. Similarly, the constitutive activation of pSTAT5 and pCRKL was diminished only in the presence of dasatinib, confirming the cell death assay results (Figure 2C).

## 3 Discussion

In this study, we identified the novel *TPR::ABL2* fusion gene by mRNA and Sanger sequencing and demonstrated its oncogenicity *in vitro* and susceptibility to ATP‐competitive TKIs currently in clinical use. Few reports have described fusion genes involving *TPR* in malignancies. The *TPR::MET* fusion gene is commonly found in gastric carcinoma, while other *TPR* fusion genes have been identified in paediatric patients with ALL and other malignancies [1, 7, 9, 10, 11]. Our findings suggest that haematological malignancies harbouring *ABL2‐r* involving the *TPR* gene may be amenable to targeted therapies, such as TKIs.

TPR is associated with the nuclear pore complex (NPC), encoding a structural component of the nuclear basket, which mediates macromolecule and mRNA export from the nucleus to the cytoplasm. While fusions involving the nucleoporin genes *NUP214* and *NUP98* have been well‐documented in haematological cancers, TPR alterations remain poorly characterised [10, 12]. TPR features a N‐terminal coiled‐coil domain containing heptad repeats and leucine zipper motifs. The coiled‐coil domain mediates the organisation of the protein into tetramers with two parallel dimers associated in an antiparallel orientation. The leucine zippers stabilise the tetrameric TPR structure by forming a hydrophobic core. The C‐terminal contains a nuclear localisation signal (NLS) [10, 12]. In the *TPR::ABL2* fusion gene identified in this study, the breakpoint resulted in the retention of the TPR N‐terminal coiled‐coil domains. Similar to *BCR::ABL1*, the oncogenic activity of *TPR::ABL2* likely results from the loss of the myristate moiety, an auto‐inhibitory element of ABL2 [5, 6]. Furthermore, the N‐terminal coiled‐coil domain of TPR may facilitate the oligomerisation of the chimeric protein and subsequent transphosphorylation of the ABL2 kinase domain, leading to the constitutive activation of signalling pathways. In *BCR::ABL1*, the oligomerisation of the chimeric protein is critical for its oncogenic activity [5]. In accordance with previous studies, primary BMMNCs and Ba/F3 cells expressing *TPR::ABL2* were sensitive to ATP‐competitive TKIs [7]. In addition, the *ABL2* breakpoint of the *TPR::ABL2* fusion gene is located at Exon 5, and the SH3 domain critical for mediating asciminib efficacy is lost. Absence of a functional SH3 domain abrogated the inhibitory effect of the allosteric inhibitor asciminib on the TPR::ABL2 oncoprotein, confirming our previous findings [7, 13, 14, 15].

Patient response to dasatinib prior to HSCT is suggestive of an effective targeted therapy for the novel *TPR::ABL2* fusion gene. Furthermore, the *TPR*::ABL2 was sensitive to multiple ATP‐competitive TKIs both in patient cells and in an in vitro cell line model. The evidence presented here supports the incorporation of TKIs into the treatment strategies for patients with *ABL2‐*rearranged ALL, improving clinical management.

## Author Contributions

E.L. designed the study, performed experiments, analysed the data, wrote the manuscript and created the figures. E.C.P., L.N.E., C.S., J.A.R., and S.L.H. performed experiments. A.S.M., M.H., M.W., D.T.Y., T.P.H., and D.L.W. provided scientific and clinical insight. All authors critically reviewed the manuscript.

## Funding

This study was undertaken with the financial support of a National Health and Medical Research Council APP2007908 and an MRFF2007441 grant. Elias Lagonik was supported by a University of Adelaide Research Scholarship. Elyse. C. Page, Laura N. Eadie and David T. Yeung were supported with fellowship funding from the Cancer Council SA's Beat Cancer Project on behalf of its donors and the State Government through the Department of Health.

## Conflicts of Interest

David T. Yeung receives research support from BMS and Novartis and Honoraria from BMS, Novartis, Pfizer, Ascentage and Amgen. Timothy P. Hughes receives research support from BMS and Novartis and Honoraria from BMS, Novartis and Fusion Pharma. Deborah L. White receives research support from BMS and Honoraria from BMS and AMGEN. The other authors declare no conflicts of interest.

## Supporting information




**Supporting Figure 1**: Immunophenotypic profile of TPR::ABL2 T‐ALL patient bone marrow mononuclear cells (BMMNCs). A) Immunophenotyping of the primary patient sample revealed that the blast population was approximately ‐73.4%. Leukaemic/blast cells B‐F) were 63% CD3 +/CD7 +, 9% CD3 +/CD7‐ and 20% CD3 −/CD7 + with negligible expression of CD34 +, CD13+, CD33+ and CD117+ (c‐kit). **Supplemental Figure 2**: Multiplex Ligation‐dependent Probe amplification analysis. The SALSA MLPA Probemix #P202, #P335, and #P383 T‐ALL oligonucleotide probes were utilised. MLPA analysis on genomic DNA revealed a heterozygous deletion of LEF1 (exon 1–4) and a homozygous deletion of CDKN2A (exon 4). Deletions are highlighted in red. **Supplemental Table 1**: Antibodies List. **Supplemental Table 2**: Materials.

## Data Availability

The data that support the findings of this study are available from the corresponding author upon reasonable request.
