# Supplementary material for: Identification of a Novel, Oncogenic and Targetable TPR::ABL2 Fusion Gene in T‐Cell Acute Lymphoblastic Leukaemia
Source: EJHaem. 2026 Mar 2;7(2):e70255. doi: 10.1002/jha2.70255 (PMC12954347; doi:10.1002/jha2.70255)
Supplement: Supplementary file 1 — Supporting Figure 1: Immunophenotypic profile of TPR::ABL2 T‐ALL patient bone marrow mononuclear cells (BMMNCs). A) Immunophenotyping of the primary patient sample revealed that the blast population was approximately ‐73.4%. Leukaemic/blast cells B‐F) were 63% CD3 +/CD7 +, 9% CD3 +/CD7‐ and 20% CD3 −/CD7 + with negligible expression of CD34 +, CD13+, CD33+ and CD117+ (c‐kit). Supplemental Figure 2: Multiplex Ligation‐dependent Probe amplification analysis. The SALSA MLPA Probemix #P202, #P335, and #P383 T‐ALL oligonucleotide probes were utilised. MLPA analysis on genomic DNA revealed a heterozygous deletion of LEF1 (exon 1–4) and a homozygous deletion of CDKN2A (exon 4). Deletions are highlighted in red. Supplemental Table 1: Antibodies List. Supplemental Table 2: Materials. [file JHA2-7-e70255-s001.docx]

**Supplemental Methods and Materials**

**Identification of a novel, oncogenic and targetable *TPR::ABL2* fusion gene in T-cell Acute Lymphoblastic Leukaemia**

**Running title:** *TPR::ABL2* in T-ALL

Elias Lagonik^1,3^, Elyse. C. Page^1,2^, Laura N. Eadie^1,2^, Caitlin Schutz^1,2^, Jacqueline A. Rehn^1,2^, Susan L. Heatley^1,2,8^, Andrew S. Moore^4^, Muirin Healy^5^, Morag Whyte^6^, Timothy P. Hughes^1,2,7^, David T. Yeung^1,2,7^, Deborah L. White^1,2,8^.

^1^Precision Cancer Medicine Theme, South Australian Health and Medical Research Institute, Adelaide, SA, Australia.

^2^Faculty of Health and Medical Sciences, The University of Adelaide, SA, Australia.

^3^Faculty of Sciences, Engineering and Technology, The University of Adelaide, SA, Australia.

^4^Oncology Service, Children’s Health Queensland Hospital, Brisbane, QLD, Australia.

^5^Paediatrics Department, Mackay Base Hospital, Mackay, QLD, Australia.

^6^Haematology Department, Queensland Children’s Hospital, Brisbane, QLD, Australia.

^7^Haematology Department, Royal Adelaide Hospital, Adelaide, SA, Australia.

^8^Australian and New Zealand Children’s Haematology-Oncology Group, Clayton, VIC, Australia.

**Compounds**

ATP-competitive Tyrosine Kinase Inhibitor imatinib (CAT# S1026), dasatinib (CAT# S1021), ponatinib (CAT# S1490), asciminib compound (CAT# S8555), and ruxolitinib (CAT# S1378) were purchased from Selleckchem. Inhibitors were dissolved in DMSO to a concentration of 10 mM and stored at -80^o^C. Imatinib was dissolved in nuclease-free H_2_O.

**Cell line maintenance**

The murine Ba/F3 cell line was cultured at 37^o^C/5% CO_2_ in RPMI media (Thermo-Fisher, Waltham, MA) supplemented with 10% Foetal Calf Serum (FCS), and 5% WEHI-3B, as a source of murine IL-3. Transduced cells were cultured without IL-3 to establish cytokine independence. When cytokine-independent, Ba/F3 cells were cultured in the absence of WEHI-3B. The HEK-293T cell line was maintained in DMEM media (Thermo-Fisher) supplemented with 10% FCS. All culture media contained 2 mM L-glutamine, 20 Units/mL penicillin, and 20 mg/mL streptomycin sulfate. Both cell lines were sourced from ATCC (Manassas, VA).

***TPR::ABL2* patient cells**

The patient’s parent/guardian provided written informed consent for sample collection and storage for future ethically approved research in accordance with the Declaration of Helsinki, including the studies presented here. Experiments were conducted in accordance with the Women’s and Children’s Health Network Human Research Ethics Committee: Approval (2023/HRE00071).

***TPR::ABL2* identification**

The *TPR::ABL2* fusion gene was identified by mRNA-sequencing, as previously described.^1^ Stranded polyA libraries were prepared using the Nugen universal Plus mRNA-seq protocol, and sequencing was performed on Illumina NextSeq500 (75bp PE reads). Gene fusions were detected using a combination of Arriba (v2.1.0)^2^, STAR-fusion (v1.10.0)^3^ and FusionCatcher (v1.33)^4^ fusion calling algorithms.

**Multiplex Ligation-dependent Probe Amplification (MLPA)**

The SALSA MLPA assay probes (#P202, #P335, and #P383; MRC-Holland) were utilised as per the manufacturer’s instructions.

**Flow cytometry in patient and murine cells**

Upon thawing, *TPR::ABL2* patient cells were incubated in IMDM media (Thermo-Fisher) supplemented with 1% BSA for 2 hours. Patient BMMNC and Ba/F3 cells were incubated for 2 hours with 5 μM of imatinib or asciminib, 100 nM dasatinib or ponatinib, or 1 μM ruxolitinib and fixed for 10 minutes with 1.6% paraformaldehyde. Cells were washed in 1x PBS and permeabilised with ice-cold 80% methanol overnight at -20^o^C. The cells were resuspended in 1x PBS, 1x PBS/1% Bovine Serum Albumin (BSA), followed by staining in the dark at room temperature for 60 minutes with pSTAT5 and pCRKL antibodies and a final wash in 1x PBS before being analysed on a BD FACSCanto^TM^ Flow cytometry system (BD Biosciences). For patient MNC phenotyping, patient cells were resuspended in RPMI supplemented with 10% FCS and stained for CD34, CD3, CD7, CD13, CD33, and CD117 (c-kit) and incubated on ice in the dark for 30 minutes. MNCs were washed with RPMI/10% FCS and analysed on a BD FACSymphony^TM^ Flow cytometry system (BD Biosciences). Antibodies are detailed in Supplemental Table 1.

**PCR amplification of *TPR::ABL2* from patient material and HAC cloning**

Amplification of the *TPR::ABL2* fusion gene from patient cells was performed in two segments (A and B). Segment A (*TPR* exon 1-23) was amplified with Primers 1 and 2. Segment B (*TPR* exon 24 to *ABL2* exon 12) was amplified utilising Primers 3 and 4 (Supplemental Table 2). **Note 1:** The red portion of these primers is complementary to the pRUFiG2 expression vector. **Note 2:** Primers 2 and 4 are complementary to each other. Segments A and B were PCR-amplified using 0.5-1 μL of cDNA, 200 μM dNTPs (Invitrogen), 0.4 μM forward/reverse primers, 1 unit of Q5 High-Fidelity polymerase, 1x Q5 Reaction Buffer, 20% Q5 high GC Enhancer (New England Biolabs, Ipswich, MA) in a 25 μL reaction. PCR-amplified segments A and B were gel-purified following the manufacturer’s protocol (QIAquick® Gel Extraction Kit, Qiagen). The purity and concentration of the PCR samples were assessed using a Qubit 2.0 fluorometer (Invitrogen). The purified PCR segments A and B (at equal DNA concentrations) were aligned and joined by a short PCR reaction (5 cycles) in the absence of primers, utilising the overlapping regions of Segment A and B **(Note 2)**. This step exploited the antibody-based hot-start technology of Platinum^TM^ SuperFi II polymerase, as per the manufacturer’s instructions. Following the short PCR reaction, Primers 1 and 3 were added, and a PCR reaction of 35 cycles was performed. The full-length *TPR::ABL2* PCR product was gel-purified according to the manufacturer’s protocol (QIAquick® Gel Extraction Kit). The full-length purified *TPR::ABL2* fusion gene was subsequently cloned into a SnaBI (1U/μL) blunt-end digested pRUFiG2 plasmid using the complementary to pRUFiG2 regions **(Note 1)** and the ligation-independent cloning method previously described here.^5^

**Plasmid preparation**

The pRUFiG2-*TPR::ABL2* (≥10 ng of DNA) was added to 50 μL of chemically competent DH5α *E. Coli* (New England Biolabs) and incubated on ice for 30 mins. The *E. Coli* were then heat-shocked for 2 mins at 42 °C and immediately placed on ice for 2 mins, followed by the addition of 0.5-1 mL of Super Optimal broth with Catabolite repression (SOC; Thermo-Fisher), media, and incubation for 1 h at 37^o^C while shaking at 200 rpm. The *E. Coli* were plated on agar plates containing ampicillin (100 μg/mL) and incubated overnight at 37^o^C. Single colonies were selected and expanded in 50 mL of Luria Broth (LB) media containing ampicillin (100 μg/mL). Plasmids were extracted utilising the technique previously described here.^6^

**Viral transduction**

Retroviral particles were produced by transient co-transfection of 6x10^5^ HEK-293T cells in 5 mL of appropriate media with 4 μg of pRUFiG2-*TPR::ABL2* plasmid and 4 μg of pEQ-eco retroviral packaging vector. The transfections were performed in Opti-MEM media (Gibco, Waltham, MA) supplemented with 3-5% Lipofectamine 3000 (Invitrogen). The transfected HEK-293T cells were confirmed to express GFP by fluorescence microscopy before the collection and filtration of the viral supernatant through a 0.45 μm filter. The viral supernatant was utilised to transduce 1x10^6^ Ba/F3 cells by spinfection at 1800 rpm for 1 hour in the presence of 4 μg/mL polybrene (Sigma Aldrich). Cells harbouring the *TPR::ABL2* fusion gene were sorted for GFP+ (>95% purity) with the BD FACSMelody^TM^ cell sorter.

**Cell death assays**

Ba/F3 cells expressing the *TPR::ABL2* fusion gene were seeded at 4x10^4^ cells/mL in a 24-well plate and treated with vehicle control (DMSO) or increasing inhibitor concentrations for 72 hours. Cells were washed with a binding buffer comprising HANK’s Balanced Salt Solution (Sigma-Aldrich, St Louis, MO), supplemented with 5 mΜ calcium chloride (Sigma-Aldrich) and 1% HEPES (Sigma-Aldrich) and stained for 20 minutes in the dark with 0.4 µL AnnexinV-PE (BD Biosciences, Franklin Lakes, NJ) and 0.04 μL 7-AAD (ThermoFisher, Waltham, MA) in 20 μL of binding buffer. The median lethal dose to kill 50% of cells (LD50) was calculated as a measure of treatment-induced cytotoxicity using GraphPad Prism software (Version 9.5.1, GraphPad Software Inc., La Jolla, CA).

**Quantification and statistical analysis**

FlowJo software (Version 10.8.1, FlowJo LLC, Ashland, OR) and GraphPad Prism software were used for experimental and statistical analysis. All experiments were performed in a minimum of biological triplicate, and graphs represent the mean with standard deviation (SD) error bars. Non-linear regression was used to calculate the LD_50_. Unpaired Student’s t-test with Welch’s correction was utilised to determine significant differences between experimental groups. Statistical significance was considered when the p-value was <0.05 (*p<0.05, **p<0.01, ***p<0.001, and ****p<0.0001).

| **Supplemental Table 1: Antibodies List** | | |
| --- | --- | --- |
| **Protein** | **Conjugate** | **Manufacturer** |
| Annexin V | PE | BD, CAT #556421 |
| 7-AAD |  | Thermofisher, CAT #A1310 |
| IgG1 | PE | BD, CAT #555749 |
| IgG1 | FITC | DC, CAT #x0927 |
| IgG2a | PE | BD, CAT #559319 |
| IgG2a | FITC | BD, CAT #555749 |
| IgG1 | A647 | Biolegend, CAT#400130 |
| IgG1 | PECy7 | BD, CAT#557872 |
| CD3 | FITC | BD, CAT #340542 |
| CD7 | PE | BD, CAT #340581 |
| CD13 | PE | BD, CAT #347837 |
| CD33 | FITC | BD, CAT #340533 |
| CD34 | A647 | BD, CAT, #343508 |
| CD117 | PECy7 | BD, CAT #339195 |
| pSTAT5 | PE | BD, CAT #612567 |
| pCRKL | PE | BD, CAT #560788 |

| **Supplemental Table 2: Materials** | |
| --- | --- |
| **Primer Sequences** | |
| Primer 1 (Forward) | 5’-AATTCAGATCTCCGCGGTACCTTTCTTCTCCGCTTCTACC-3’ |
| Primer 2 (Reverse) | 5’-CCATGCTCTCTATGGCTCTTC-3’ |
| Primer 3 (Reverse) | 5’-CTCGAGGATCTCATATGTACCTACCTCTGCACCACATCAC-3’ |
| Primer 4 (Forward) | 5’-GAAGAGCCATAGAGAGCATGG-3’ |


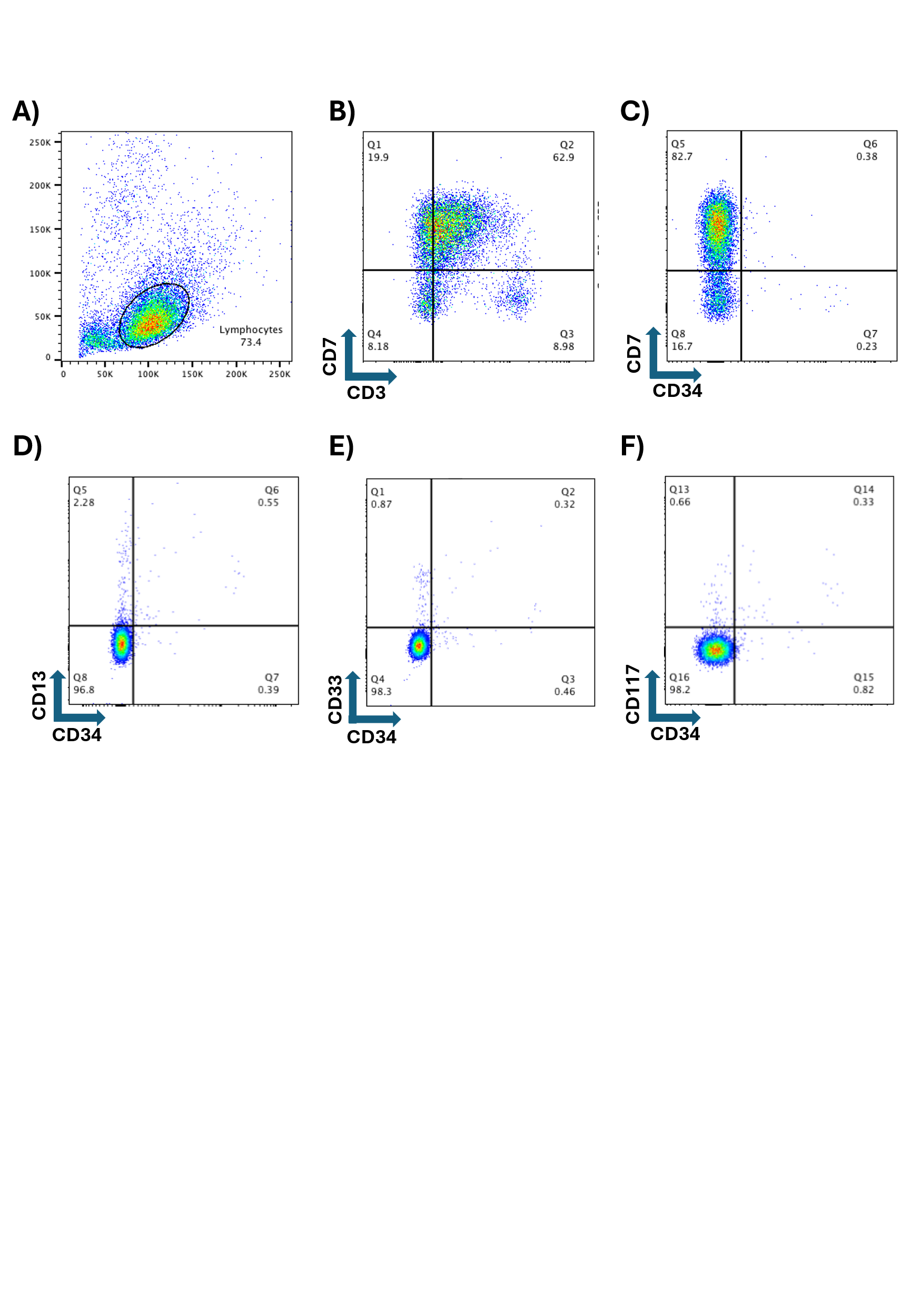


**Supplemental Figure 1: Immunophenotypic profile of *TPR::*ABL2 T-ALL patient bone marrow mononuclear cells (BMMNCs).** A) Immunophenotyping of the primary patient sample revealed the blast population was approximately ~73.4%. Leukaemic/blast cells B-F) were 63% CD3+/CD7+, 9% CD3+/CD7- and 20% CD3-/CD7+ with negligible expression of CD34+, CD13+, CD33+ and CD117+ (c-kit).


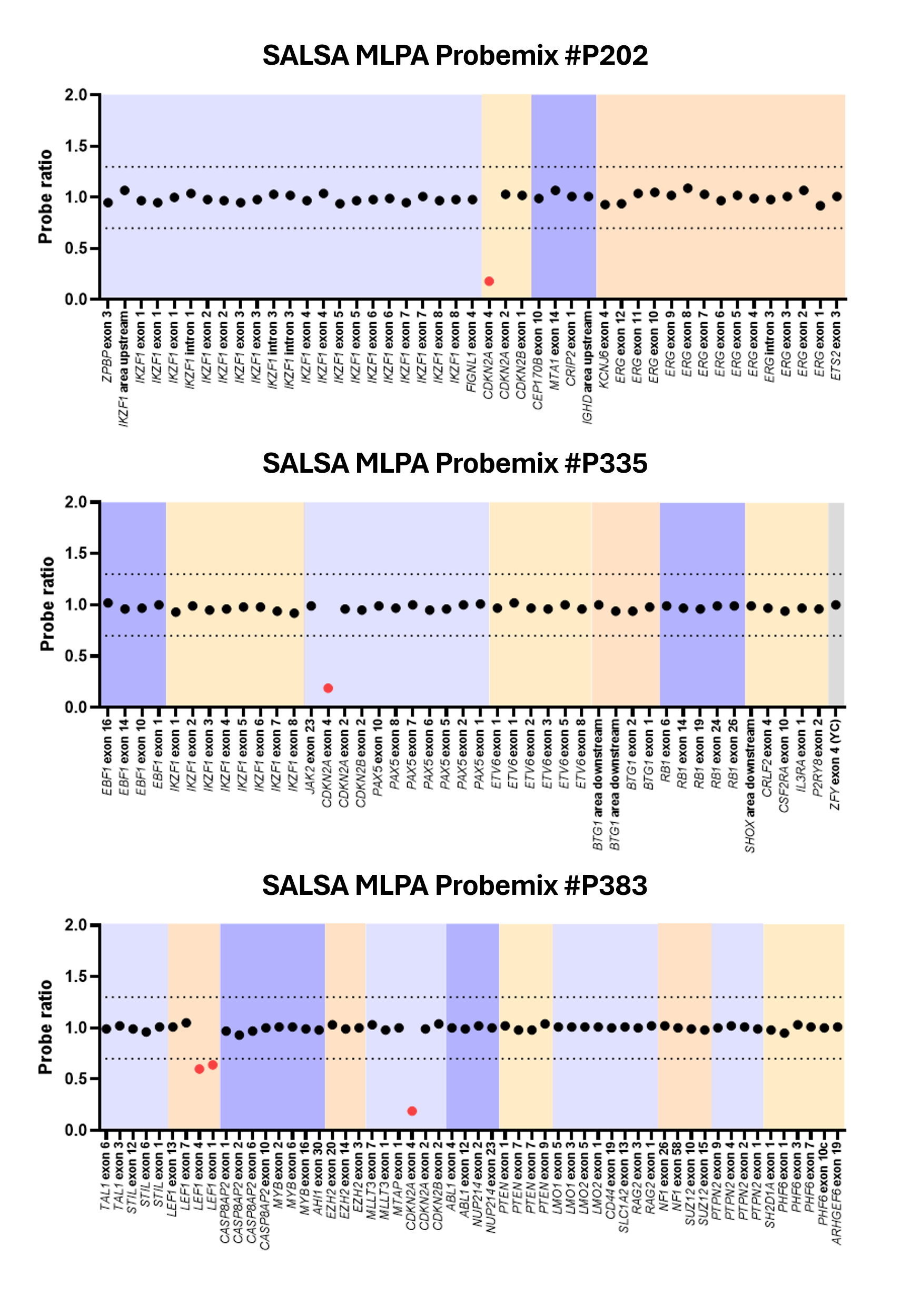


**Supplemental Figure 2: Multiplex Ligation-dependent Probe amplification analysis.** The SALSA MLPA Probemix #P202, #P335, and #P383 T-ALL oligonucleotide probes were utilised. MLPA analysis on genomic DNA revealed a heterozygous deletion of *LEF1* (exon 1-4) and a homozygous deletion of CDKN2A (exon 4). Deletions are highlighted in red.

**References**

1. Rehn J, Mayoh C, Heatley SL, et al. RaScALL: Rapid (Ra) screening (Sc) of RNA-seq data for prognostically significant genomic alterations in acute lymphoblastic leukaemia (ALL). *PLOS Genetics*. 2022;18(10):e1010300.

2. Uhrig S, Ellermann J, Walther T, et al. Accurate and efficient detection of gene fusions from RNA sequencing data. *Genome Res*. 2021;31(3):448-460.

3. Haas BJ, Dobin A, Stransky N, et al. STAR-Fusion: Fast and Accurate Fusion Transcript Detection from RNA-Seq. *bioRxiv*. 2017:120295.

4. Nicorici D, Şatalan M, Edgren H, et al. <strong>FusionCatcher</strong> – a tool for finding somatic fusion genes in paired-end RNA-sequencing data. *bioRxiv*. 2014:011650.

5. Tan L, Strong EJ, Woods K, West NP. Homologous alignment cloning: a rapid, flexible, and highly efficient general molecular cloning method. *PeerJ*. 2018;6:e5146.

6. Pronobis MI, Deuitch N, Peifer M. The Miraprep: A Protocol that Uses a Miniprep Kit and Provides Maxiprep Yields. *PLoS One*. 2016;11(8):e0160509.
